# Economic Burden of Hypoglycemia in Patients with Type 2 Diabetes Mellitus from Korea

**DOI:** 10.1371/journal.pone.0151282

**Published:** 2016-03-14

**Authors:** Gyuri Kim, Yong-ho Lee, Mi Hye Han, Eui-Kyung Lee, Chong Hwa Kim, Hyuk Sang Kwon, In Kyung Jeong, Eun Seok Kang, Dae Jung Kim

**Affiliations:** 1 Department of Internal Medicine, Yonsei University College of Medicine, Seoul, Republic of Korea; 2 Graduate School, Yonsei University College of Medicine, Seoul, Republic of Korea; 3 Market Access, Merck Sharp & Dohme, Seoul, Republic of Korea; 4 Pharmaceutical Policy & Outcomes Research, School of Pharmacy, Sungkyunkwan University, Seoul, Republic of Korea; 5 Department of Internal Medicine, Sejong General Hospital, Bucheon, Republic of Korea; 6 Department of Internal Medicine, College of Medicine, The Catholic University of Korea, Seoul, Republic of Korea; 7 Department of Internal Medicine, Kyung Hee University School of Medicine, Seoul, Republic of Korea; 8 Department of Endocrinology and Metabolism, Ajou University School of Medicine, Suwon, Republic of Korea; Baylor College of Medicine, UNITED STATES

## Abstract

**Background:**

Hypoglycemia is a very serious complication in patients with type 2 diabetes mellitus (T2DM) and affects the economic burden of treatment. This study aims to create models of the cost of treating hypoglycemia in patients with T2DM based upon physician estimates of medical resource usage.

**Methods:**

Using a literature review and personal advice from endocrinologists and emergency physicians, we developed several models for managing patients with hypoglycemia. The final model was approved by the consulting experts. We also developed 3 unique surveys to allow endocrinologists, emergency room (ER) physicians, and primary care physicians to evaluate the resource usage of patients with hypoglycemia. Medical costs were calculated by multiplying the estimated medical resource usage by the corresponding health insurance medical care costs reported in 2014.

**Results:**

In total, 40 endocrinologists, 20 ER physicians, and 30 primary care physicians completed the survey. We identified 12 types of standard medical models for secondary or tertiary hospitals and 4 for primary care clinics based on the use of ER, general ward, or intensive care unit (ICU) and patients’ status of consciousness and self-respiration. Estimated medical costs per person per hypoglycemic event ranged from $17.28 to $1,857.09 for secondary and tertiary hospitals. These costs were higher for patients who were unconscious and for those requiring ICU admission.

**Conclusion:**

Hypoglycemia has a substantial impact on the medical costs and its prevention will result in economic benefits for T2DM patients and society.

## Introduction

Type 2 diabetes mellitus (T2DM) is one of the most serious health problems worldwide, and its acute and chronic complications have substantial clinical impact on healthcare and society [1]. The global population of diabetes mellitus is expected to grow from 171 million in 2000 to over 366 million people by 2030, with T2DM comprising 90% of all diabetes cases worldwide [2]. The largest proportion of treatment costs for diabetes is attributable to its various complications [3,4]. Because of therapeutic efforts to maintain target HbA1c, an increasing number of T2DM patients currently live with the risk of hypoglycemia [5]. Hypoglycemia is one of the severe and undesirable side effects of aggressive glycemic control, and the cost of managing a severe hypoglycemic episode is comparable to that of nephropathy and is higher than that of retinopathy [6]. In the ADVANCE study, during a 5-year median follow-up period, 44.6% of T2DM patients had minor hypoglycemia and 2.1% had at least 1 severe hypoglycemic event [7]. The incidence of hypoglycemia in T2DM patients taking oral antidiabetic drugs is 16%-39% and 35.8% in the U.S. and Europe and Asia-Pacific region, respectively [8–10]. The risk of severe hypoglycemia was 2 times higher in patients receiving insulin compared to those receiving oral therapies without secretagogues. In addition, age, female gender, longer diabetes duration, presence of diabetic retinopathy or neuropathy, and number of glucose-lowering drugs increased the risk of symptomatic hypoglycemia [11]. Also, hypoglycemia itself has been associated with numerous undesirable events with neurological impairment such as cognitive ability, inpatient mortality, and longer hospital stays [12,13]. Moreover, in previous studies, treatment costs for hypoglycemia were typically calculated for severe hypoglycemic episodes alone. These costs had reached over $3,282.30, which is similar to costs for chronic complications and represents an enormous economic impact on the healthcare system [6]. However, cost estimation for treating hypoglycemia is complicated because it includes both direct and indirect medical costs associated with each type of episode, which can drastically vary based on severity of hypoglycemic symptoms, treatment setting (hospitals or primary care clinics), and frequency of episodes. Direct medical costs involve costs paid by outpatients and inpatients at medical institutions and pharmacies for direct treatment of hypoglycemia [14]. However, few studies have investigated direct medical costs for treating hypoglycemia in real-world clinical settings, accounting for variance in treatment model, setting (i.e., primary care clinics, outpatients clinics in secondary and tertiary hospitals, or emergency rooms [ERs]), and hospitalization in general wards or intensive care units (ICUs).

Therefore, the present study aimed to (1) create models of the cost for treating hypoglycemia based on physician estimates of resource usage for T2DM patients in outpatient or inpatient (including ER or ICU) settings of secondary or tertiary hospitals as well as primary care clinics and (2) estimate the medical costs associated with treating hypoglycemia based on these models.

## Materials and Methods

### Development of a standard medical model

A hypoglycemic event is defined as a blood glucose level <70 mg/dL or an episode with symptoms of low blood glucose levels. The first draft version of a standard medical model for treating hypoglycemia in patients with T2DM was developed based on local and foreign literature review. In Korea, there are largely 3 categories of health care system. Firstly, primary health care is the first stage of health care for outpatient care and usually health promotion centers and primary care clinics offer this kind of health care. In addition, secondary and tertiary hospital provide professional health care from specialists including both inpatient and outpatient care. Secondary hospital needs to have at least 4 departments with professional doctors and at least 30 inpatient beds. Tertiary hospital needs to have a full complement of departments and specific sub-specialty care with professional doctors and at least more than 500 beds. A patient is referred from primary health care to a secondary or tertiary hospital when further treatment and intensive care facilities are required. The second version was developed by obtaining one-on-one advice from 5 endocrinologists working at secondary and tertiary hospitals and 2 emergency physicians to represent medical practices in the ER. The final version was developed by revising and complementing the draft model based on the consensus of the expert advisory council members who confirmed the general medical resources used for treating hypoglycemia.

### Development of a physician questionnaire

We developed the draft version of a questionnaire to investigate medical resource usage through literature review and the experts’ advice regarding the standard medical model for treating hypoglycemia. To confirm the validity of the questionnaire, we performed a preliminary survey including 4 endocrinologists, 2 emergency physicians, and 3 primary care physicians. The number of physicians was equivalent to 10% of the physicians intended for enrollment. Based on one-on-one interview and feedback received from these physicians, we revised and complemented the preliminary questionnaire and subsequently created 3 final questionnaires for endocrinologists, emergency physicians from secondary and tertiary hospitals, and primary care physicians, respectively (S1 File). We specified each question in the questionnaire to calculate each proportion per step in each type of the standard medical model according to physician (primary physician, endocrinologist, and emergency physician) and hospital setting (primary clinic and secondary or tertiary hospital), so the incidence of type of standard medical model was calculated by multiplying each proportion by step.

### Evaluation of medical resources for treating hypoglycemia

We evaluated the medical resource usage for treating hypoglycemia among T2DM patients using a one-on-one interview-style questionnaire survey of 40 endocrinologists, 20 emergency physicians from secondary and tertiary hospitals, and 30 primary care physicians. The questionnaire was designed to estimate average medical resource usage based on the respondent’s personal experience rather than a retrospective chart review. The medical resource usage included the incidence of hypoglycemia by the type of standard medical model and the prescription rate of each medical resource, such as laboratory and imaging tests and treatments including infusions of dextrose in water, monitoring, catheter insertion, intubation, and cardiopulmonary resuscitation. Responses were stratified by treatment setting (ER, general ward, or ICU), patient’s status of consciousness and self-respiration, and treatment department (endocrinology or ER). For primary care clinics, including departments of internal medicine, endocrinology, and family medicine, only outpatient clinic data were included because patients with severe symptoms rarely visit such clinics.

### Calculation of medical costs

Medical costs per case and type from the standard medical model were calculated using survey results of medical resource usage and for each group (outpatient/ER/inpatient/ICU) by multiplying the investigated medical resource usage by the corresponding 2014 health insurance medical care expenses (S1 Table). All costs were expressed in U.S. dollars ($) based on the final exchange rate of July 2015. Medical costs excluded drug costs and were calculated by adding the patient’s and insurer’s shares of medical costs for health insurance benefits, including certain uninsured expense items, which insurance do not cover so that a patient pays a total amount of the expense. The cost of magnetic resonance imaging (MRI) performed for patients who visited ERs due to hypoglycemia was included as an uninsured expense item. For uninsured expense items, uninsured expenses were based on the Health Insurance Review & Assessment Service (HIRA), and the average value of each item classified by type of institution was calculated and applied. Clinically invalid values, as determined by the expert advisory council, were handled as missing values, and average costs of relevant items were imputed in place of missing values. Moreover, for undetermined responses due to lack of understanding a survey question, cost was calculated by changing to a valid value. Costs for medical resources included in the survey response for other items were also calculated.

### Statistical analyses

All continuous variables were expressed as means (standard deviations [SD]), and categorical variables were expressed as frequencies with percentages. Differences were analyzed using ANOVA. P<0.05 represented a statistically significant difference. Statistical analyses were performed using the PASW Statistics software, version 20.0 for Windows (SPSS Inc., Chicago, IL, U.S.).

## Results

### Development of standard medical model

**Fig 1:**
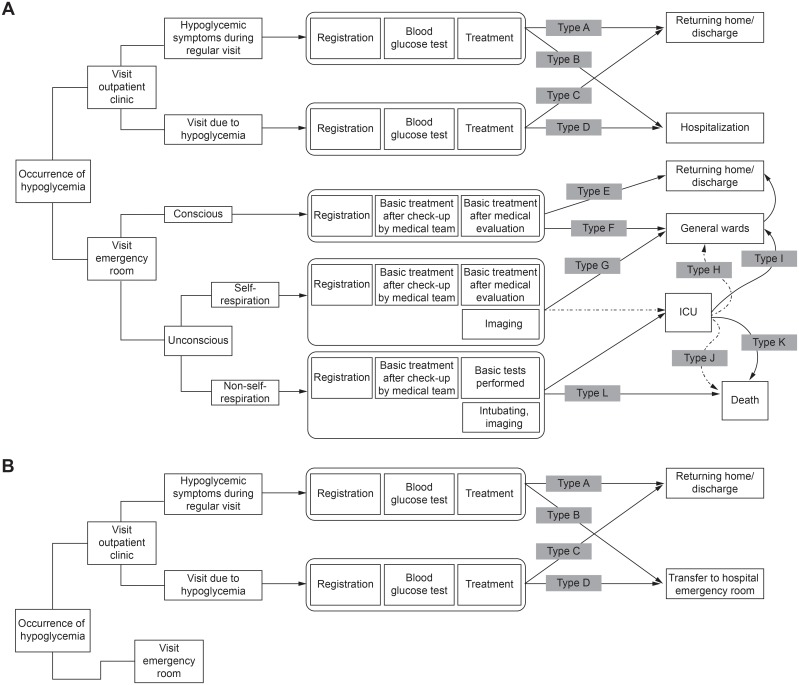
Standard medical model for treating hypoglycemia in patients with type 2 diabetes mellitus. (A) Secondary and tertiary hospitals. (B) Primary care clinics. Type H: an unconscious patient who could breathe independently and was hospitalized in the intensive care unit and general ward after visiting the emergency room and receiving treatment; Type I: an unconscious patient who could not breathe independently and was hospitalized in the intensive care unit and general ward after visiting the emergency room and receiving treatment; Type J: an unconscious patient who could breathe independently, visited the emergency room, received treatment, was hospitalized in the intensive care unit and subsequently died; Type K: an unconscious patient who could not breathe independently, visited the emergency room, received treatment, was hospitalized in the intensive care unit, and subsequently died. ICU, intensive care unit.

**Fig 2:**
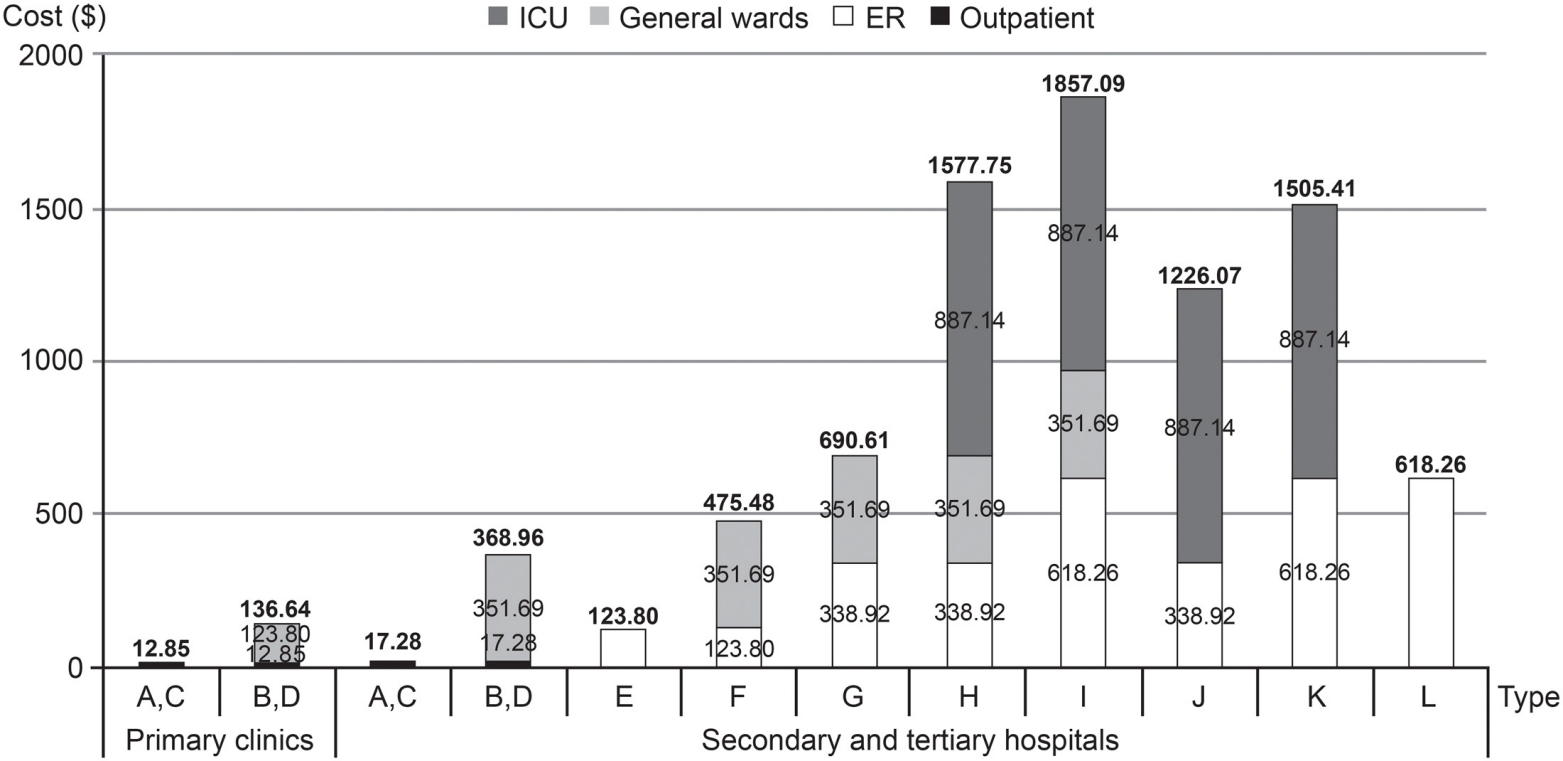
Medical costs for treating hypoglycemia in secondary and tertiary hospitals and primary clinics according to model type. ER, emergency room; ICU, intensive care unit.

We created 12 standard medical models for secondary or tertiary hospitals (Fig 1) and 4 standard medical models for primary care clinics (Fig 2); these were developed through local and foreign literature review and expert consensus. As patients with hypoglycemia might visit an ER or outpatient clinic, patients were categorized according to the department they visited. Moreover, as we aimed to develop a standard medical model for treating hypoglycemia, we excluded asymptomatic patients from those who visited any department for regular check-ups. Patients visiting outpatient clinics were categorized into 2 types based on the outpatient flow: patients with hypoglycemic symptoms during regular visits (Type A or B) and patients visiting hospitals due to a hypoglycemic event (Type C or D). Furthermore, because hospitalization may have been required after a visit to an outpatient clinic, flow from outpatient to inpatient care was included (Type B or D). For the ER treatment model, major factors for classification were level of consciousness, self-respiration ability, and treatment at a general ward, ER, or ICU. Among patients who visited ERs, conscious patients were discharged home or were admitted to general wards and then discharged (Type E or F). Unconscious but self-breathing patients who visited ERs were discharged from general wards (Type G), discharged from ICUs via general wards (Type H), or died after ICU admission (Type J). Type G also includes unconscious patients who were hospitalized in general wards shortly because they stably recovered after dextrose infusion. Unconscious patients who could not breathe independently had the most severe symptoms and were either treated in ICUs or died (Types I and K or Type L). For patients admitted to ICUs, improvements in condition often resulted in change of hospitalization status; accordingly, we created a flow chart of their general ward status (Type I). Furthermore, as patients might die in ICUs due to lack of improvement, we created a flow chart of their status from ICUs to death (Type K).

### Calculation of medical costs

On an average, 650.0 patients (mean age, 57.2 years; mean T2DM duration, 7.7 years; men, 45.0%) made regular visits to an endocrinologist per month at outpatient clinics of secondary and tertiary hospitals, and 433.7 patients (mean age, 56.3 years; mean T2DM duration, 6.9 years; men, 43.5%) made regular visits to a primary care physician per month at primary care clinics (S2 Table). There was no significant correlation between costs for treating hypoglycemia and the duration of physicians’ clinical experience among the 90 physicians (P = 0.065). Moreover, the 7 metropolitan regions surveyed showed no significant differences in the incidence of hypoglycemia (P = 0.739). Furthermore, no differences were observed in the duration of hospitalization between secondary and tertiary hospitals. Tables 1 and 2 present the medical costs for treating hypoglycemia in secondary and tertiary hospitals and primary care clinics based on the model type. Among hypoglycemic T2DM patients visiting secondary and tertiary hospitals, the most common model type observed was Type A (non-severe hypoglycemia; 47.01%), which included patients discharged home after receiving treatment for hypoglycemic symptoms during a regularly scheduled visit at an outpatient clinic, followed by Type G (11.31%), which included self-breathing unconscious patients who were hospitalized in general wards after visiting an ER. In primary care clinics, 82.68% of hypoglycemic patients presented as Type A. The estimated medical costs per hypoglycemic event ranged from $17.28 (Type A or C) to $1,857.09 (Type I) for secondary and tertiary hospitals and from $12.85 (Type A or C) to $136.64 (Type B or D) for primary care clinics. S1A and S1B Fig present the specific incidence rates of hypoglycemia in T2DM patients who visited secondary and tertiary hospitals and primary care clinics according to each step in the standard medical model. Moreover, Tables A-C in S3 and S4 Tables present survey responses and the calculation of costs for treating hypoglycemia in T2DM patients who visited secondary or tertiary hospitals according to the model type and treatment setting (outpatient, ER, general ward, and ICU). Medical costs per visit to an outpatient clinic, per admission to a general ward, and per admission to an ICU were calculated as $17.28, $351.69, and $887.14 per person per episode, respectively (P<0.001). For medical costs incurred in ERs, the per visit cost for a conscious patient, an unconscious but self-breathing patient, and an unconscious and non-self-breathing patient were calculated as $123.80, $338.92, and $618.26 per person per episode, respectively (P<0.001). Costs of imaging studies, including computed tomography (CT) or MRI, accounted for the largest proportion of total costs in treating unconscious patients in ERs.

**Table 1:** Medical costs for treating hypoglycemia at secondary and tertiary hospitals according to model type.

Type	Definition	Medical cost ($)
A (47.01%)	Costs incurred by a patient who returned home after receiving treatment for hypoglycemic symptoms during a regularly scheduled outpatient clinic visit	17.28
B (8.77%)	Costs incurred by a patient who was hospitalized after receiving treatment for hypoglycemic symptoms during a regularly scheduled outpatient clinic visit	368.96
C (10.57%)	Costs incurred by a patient who returned home after visiting an outpatient clinic due to hypoglycemic symptoms	17.28
D (3.15%)	Costs incurred by a patient who was hospitalized after visiting an outpatient clinic due to hypoglycemic symptoms	368.96
E (5.61%)	Costs incurred by a conscious patient who returned home after visiting the emergency room and receiving treatment	123.80
F (4.31%)	Costs incurred by a conscious patient who was hospitalized in the general ward after visiting the emergency room and receiving treatment	475.48
G (11.31%)	Costs incurred by an unconscious patient who could breathe independently and was hospitalized in the general ward after visiting the emergency room and receiving treatment	690.61
H (8.75%)	Costs incurred by an unconscious patient who could breathe independently and was hospitalized in the intensive care unit and general ward after visiting the emergency room and receiving treatment	1,577.75
I (0.28%)	Costs incurred by an unconscious patient who could not breathe independently and was hospitalized in the intensive care unit and general ward after visiting the emergency room and receiving treatment	1,857.09
J (0.17%)	Costs incurred by an unconscious patient who could breathe independently, visited the emergency room, received treatment, was hospitalized in the intensive care unit and subsequently died	1,226.07
K (0.01%)	Costs incurred by an unconscious patient who could not breathe independently, visited the emergency room, received treatment, was hospitalized in the intensive care unit, and subsequently died	1,505.41
L (0.06%)	Costs incurred by an unconscious patient who could not breathe independently and who died after visiting the emergency department	618.26

**Table 2:** Medical costs for treating hypoglycemia at primary care clinics.

Type	Definition	Medical cost ($)
A (82.68%)	Costs incurred by a patient who returned home after receiving treatment for hypoglycemic symptoms during a regularly scheduled outpatient clinic visit	12.85
B (0.28%)	Costs incurred by a patient who was transferred to a larger institution's emergency room after receiving treatment for hypoglycemic symptoms during a regularly scheduled outpatient clinic visit	136.64
C (15.62%)	Costs incurred by a patient who returned home after visiting an outpatient clinic and receiving treatment due to hypoglycemia symptoms	12.85
D (1.42%)	Costs incurred by a patient who was transferred to a larger institution's emergency room after visiting an outpatient clinic due to hypoglycemia symptoms	136.64

## Discussion

In the present study, we developed standard medical models for treating hypoglycemia in patients with T2DM and consequently identified 12 model types for secondary or tertiary hospitals and 4 model types for primary care clinics based on the use of ER, general ward, or ICU and patients’ status of consciousness and self-respiration. The estimated medical cost per hypoglycemic event ranged from $17.28 to $1,857.09 for secondary and tertiary hospitals and from $12.85 to $136.64 for primary care clinics. Patients with non-severe hypoglycemia mostly visited primary care or outpatient clinics, and per visit costs associated with these treatment types were markedly lesser compared to those who were admitted; however, non-severe hypoglycemia could be associated with greater overall costs due to its high incidence (69.53%) in secondary and tertiary hospitals. Moreover, severe hypoglycemic patients who were unconscious or admitted to an ICU represented a relatively high proportion (20.63%) of cases in secondary and tertiary hospitals and revealed considerably high costs per episode.

In the present study, standard medical model Type I—an unconscious patient with severe hypoglycemia who could not breathe independently and was hospitalized in an ICU and general ward after visiting an ER and narrowly discharged—accounted for only 0.28% of cases in secondary and tertiary hospitals but was associated with costs as high as $1,857.09 per person per episode. Moreover, from an social and economic viewpoint, even after recovery from severe hypoglycemia, the risk of accelerated dementia, repeated hypoglycemic events due to hypoglycemia unawareness, and subsequent fear of hypoglycemia associated with obstacles for achieving glycemic goals could be responsible for its enormous unmeasured burdens [12]. Conscious patients visiting ERs who did not die or require admission to an ICU and recovered safely incurred markedly lower costs compared with unconscious patients. In addition, among hypoglycemic patients visiting secondary and tertiary hospitals, although a majority of patients (69.53%) had visited outpatient clinics with mild hypoglycemia, unconscious patients who visited ER and could breathe independently accounted for a relatively large proportion as well (20.23%) with high associated costs. As patients who have comorbidities such as renal disease, cardiovascular disease, or pulmonary problems treat for hypoglycemia may require hospitalization longer or need intensive care, it could confound the cost of hospitalization [15,16]. However, we focused on the treatment for hypoglycemia alone so, we estimated the cost of medical resource usage for hypoglycemia, at most. Moreover, hypoglycemia treatment in outpatient clinics at secondary or tertiary hospitals was more costly compared to the treatment cost at primary care clinics. A previous study reported that the cost of hypoglycemia depends on the severity of the episode: for example, the annual cost per patient per episode increased from $366.20 for cases of moderate hypoglycemia, wherein a patient seeks medical attention for hypoglycemia but is not admitted overnight to a hospital, to $3180.33 for severe hypoglycemic events requiring hospitalization [17]. Ha WC et al. reported that the average medical cost incurred for severe hypoglycemia ranged from $135.50 to $1,391 in Korea, with costs for hospitalization and brain imaging comprising the largest proportion of total costs [18]. However, no study on medical costs has been conducted for each specific type after admission to a hospital, such as ICU care. Of note, the present study demonstrates that estimation of direct medical costs for hypoglycemia, particularly for cases previously defined as severe hypoglycemia requiring hospitalization, varies considerably depending on the details of treatment settings, consciousness, and ability to breathe independently. In addition, we presented differences of incidence and costs by type for secondary and tertiary hospitals, and primary care clinics.

The present study has several distinguishing strengths. For development of standard medical models of hypoglycemia, we used an incidence-based approach in relation to all existing cases in primary care clinics and secondary or tertiary hospitals, along with the possible treatment approaches, via the consensus of an expert advisory council. Therefore, we were able to identify and involve a series of specific, step-by-step treatment processes only for hypoglycemia in terms of real-world clinical practice. Based on these models, we could estimate the costs, not only for each step of a model type but also total costs per type. We also demonstrated representative results derived from a large population in 7 different metropolitan areas from a total of 90 specialized experts with extensive clinical practical experience.

The present study has several limitations. First, in the questionnaire, data were recorded based on the respondent’s personal experience rather than a retrospective chart review. Therefore, there might be some recall bias in the data reported in terms of incidence of medical resource usage. However, data were collected from 90 medical experts from various specialties and extensive clinical experience: the 40 endocrinologists, 20 emergency physicians, and 30 primary care physicians had a mean clinical experience of 13.9, 12.7, and 20.0 years, respectively. Second, for medical resource usage, although laboratory and imaging tests and treatments were included, drug costs were not included in the calculated medical costs; hence, the actual costs were probably underestimated. Last, we could not estimate the overall annual direct medical costs for hypoglycemia in Korea, which could have been calculated only by knowing the exact prevalence of hypoglycemia. Recently, the prevalence of symptomatic hypoglycemia in patients with T2DM in Korea was reported as 39.0%, but additional studies are warranted to determine the incidence of hypoglycemia for accurate estimation [9]. Moreover, we could not use real patients and assess real costs using National Health Insurance Service (NHIS) database, because it was not able to solely assess the real medical costs for treating hypoglycemia due to mixed medical costs including costs for treating other underlying disease, comorbidities, or adverse effects. Also, its discrepancy between diagnosis of individuals in real-practice and recorded in the claim database could show inconsistent and inaccurate estimation for hypoglycemia. Therefore, we assessed medical cost by various model types and focused on the costs only for treating hypoglycemia including laboratory and imaging tests and treatments based upon physician estimates of medical resource usage.

## Conclusion

In conclusion, we developed standard medical models for treating hypoglycemia in patients with T2DM: 12 model types for secondary or tertiary hospitals and 4 model types for primary care clinics. In addition, we evaluated medical resource usage and estimated direct medical costs per hypoglycemic event in order to estimate the real practical patterns and economic burden of hypoglycemia. The direct medical costs for hypoglycemia were particularly high for patients who were unconscious upon presentation and for those admitted to ICUs. It is clear that hypoglycemic events among patients with T2DM are associated with a considerable financial cost, and thus, prevention of these events can reduce the overall healthcare expenditure incurred by such patients. Therefore, glycemic control strategies and therapeutic regimens must be carefully selected with individualized patient considerations to prevent hypoglycemia in patients with T2DM.

## Supporting Information

S1 Fig Incidence of hypoglycemia in patients with type 2 diabetes mellitus by the type of standard medical model. (A) Secondary and tertiary hospitals. (B) Primary care clinics.
(TIF)

S1 File Survey questionnaire for hospital and primary care clinic use. (A) Department of endocrinology in secondary and tertiary hospitals. (B) Department of emergency medicine in secondary and tertiary hospitals. (C) Primary Care Clinic Use.
(DOCX)

S1 Table Cost items specified in the 2014 health insurance medical care expenses. (DOCX)

S2 Table Characteristics of physicians and patients with type 2 diabetes mellitus. (DOCX)

S3 Table Results of the study of medical resources usage. (A) Department of Endocrinology in secondary and tertiary hospitals. (B) Department of Emergency Medicine in secondary and tertiary hospitals. (C) Primary care clinics.
(DOCX)

S4 Table Hypoglycemia treatment costs in type 2 diabetes mellitus patients who visited secondary and tertiary hospitals. (DOCX)
