# Supplementary figures and images for: Economic Burden of Hypoglycemia in Patients with Type 2 Diabetes Mellitus from Korea
Source: PLoS One. 2016 Mar 14;11(3):e0151282. doi: 10.1371/journal.pone.0151282 (PMC4790854; doi:10.1371/journal.pone.0151282)

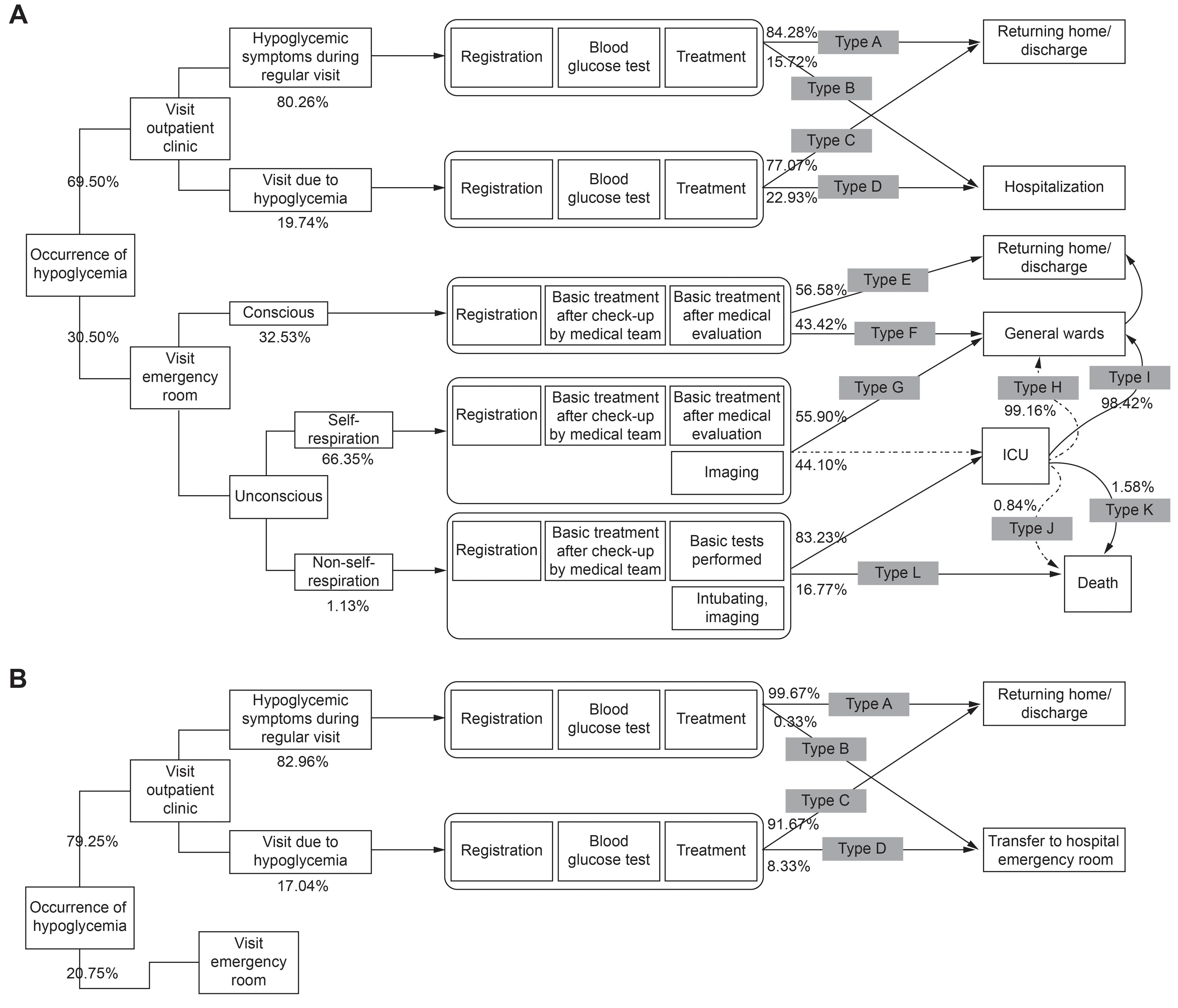

Supplement: S1 Fig — (A) Secondary and tertiary hospitals. (B) Primary care clinics. (TIF) [file pone.0151282.s001.tif]
