# Supplementary material for: Economic Burden of Hypoglycemia in Patients with Type 2 Diabetes Mellitus from Korea
Source: PLoS One. 2016 Mar 14;11(3):e0151282. doi: 10.1371/journal.pone.0151282 (PMC4790854; doi:10.1371/journal.pone.0151282)
Supplement: S1 File — (A) Department of endocrinology in secondary and tertiary hospitals. (B) Department of emergency medicine in secondary and tertiary hospitals. (C) Primary Care Clinic Use. (DOCX) [file pone.0151282.s002.docx]

**S1 File. Survey questionnaire for hospital and primary care clinic use.**

**(A) Department of endocrinology in secondary and tertiary hospitals.**

**Study of the cost of care for hypoglycemia in patients with type 2 diabetes in South Korea**

| This study aims to investigate hypoglycemia in patients with type 2 diabetes in South Korea.  In addition to verifying the incidence of hypoglycemia in patients with type 2 diabetes, the extra cost for patients with hypoglycemia will also be calculated.  The incidence of hypoglycemia by treatment type and the use of medical resources for actual treatment will be investigated.  This investigation will not be used in any form other than for research purposes, and the privacy of the information will be maintained. |
| --- |

1. **General characteristics**
2. In which region is your current place of work located?

| Metropolitan Area/Province | City/County/District |
| --- | --- |
| □ ① Seoul | □ ① ( ) City |
| □ ② ( ) Metropolitan Area | □ ② ( ) County |
| □ ③ ( ) Province | □ ③ ( ) District |

1. At which type of institution do you currently work?

□① Tertiary hospital hospital

□② Secondary hospital

□③ Primary care clinic

□④ Other ( )

1. Does your current place of work have an emergency medical center?

| □ ① Yes | □ ② No |
| --- | --- |

1. How many years of clinical experience do you have? ( years)
2. What is your specialty?

□ ① Endocrinology

□ ② Emergency medicine

□ ③ General medicine

□ ④ Family medicine

□ ⑤ Other ( )

1. What is the average number of patients with type 2 diabetes that you treat per month?

( persons)

1. What percentage of patients with type 2 diabetes that you treat are of each sex?

| Men | Women |
| --- | --- |
| ( %) | ( %) |

1. What is the average age of the patients with type 2 diabetes that you treat?

□ ① <30 years □ ② 30–39 years

□ ③ 40–49 years □ ④ 50–59 years

□ ⑤ 60–69 years □ ⑥ 70–79 years □ ⑦ ≥80 years

1. What is the average duration of type 2 diabetes for the patients that you treat?

( years)

**II. Use of outpatient services**

Please respond based on your own clinical experience with treating outpatients with hypoglycemia.

1. How many patients with type 2 diabetes visited regularly as outpatients in the last month?

( persons)

1. Of the patients with type 2 diabetes who visited regularly as outpatients in the last month, how many patients had symptoms of hypoglycemia or blood glucose levels <70 mg/dL at the time of the visit?

( persons)

1. Of the patients with type 2 diabetes who visited regularly as outpatients in the last month, how many patients were hospitalized later for hypoglycemia?

( persons)

1. How many outpatients visited in the last month because of hypoglycemia?

( persons)

1. Of the patients with type 2 diabetes who visited as outpatients because of hypoglycemia in the last month, how many patients were hospitalized later for hypoglycemia?

( persons)

1. Which of the following medical resources were prescribed for each outpatient, and how often was each resource prescribed, on average?

Please record the names and frequencies of additional tests or treatments in the ‘Other’ categories. Please provide detailed responses, e.g., liver function tests (AST, ALT, ALP, GGT, bilirubin), kidney function tests (Blood urea nitrogen, Serum creatinine, GFR, creatinine clearance).

|  | Medical Resource | Frequency |
| --- | --- | --- |
| Test | Blood sugar test | ( %, times)/visit |
|  | HbA1c | ( %, times)/visit |
|  | Glucose | ( %, times)/visit |
|  | Other 1 ( ) | ( %, times)/visit |
|  | Other 2 ( ) | ( %, times)/visit |
|  | Other 3 ( ) | ( %, times)/visit |
| Treatment | Dextrose in water 50% ( ml) | ( %, times)/visit |
|  | Dextrose in water 10% ( ml) | ( %, times)/visit |
|  | Other 1 ( ) | ( %, times)/visit |
|  | Other 2 ( ) | ( %, times)/visit |
|  | Other 3 ( ) | ( %, times)/visit |

ALT, Alanine Aminotransferase; ALP, Alkaline phosphatase; AST, Aspartate Aminotransferase; GFR, Glomerular filtration rate; GGT, gamma glutamyl transferase

**(B) Department of emergency medicine in secondary and tertiary hospitals.**

**Study of the cost of care for hypoglycemia in patients with type 2 diabetes in South Korea**

| This study aims to investigate hypoglycemia in patients with type 2 diabetes in South Korea.  In addition to verifying the incidence of hypoglycemia in patients with type 2 diabetes, the extra cost for patients with hypoglycemia will also be calculated.  The incidence of hypoglycemia by treatment type and the use of medical resources for actual treatment will be investigated.  This investigation will not be used in any form other than for research purposes, and the privacy of the information will be maintained. |
| --- |

1. **General characteristics**
2. In which region is your current place of work located?

| Metropolitan Area/Province | City/County/District |
| --- | --- |
| □ ① Seoul | □ ① ( ) City |
| □ ② ( ) Metropolitan Area | □ ② ( ) County |
| □ ③ ( ) Province | □ ③ ( ) District |

1. At which type of institution do you currently work?

□① Tertiary hospital hospital

□② Secondary hospital

□③ Primary care clinic

□④ Other ( )

1. How many years of clinical experience do you have? ( years)
2. What is your specialty?

□ ① Endocrinology

□ ② Emergency medicine

□ ③ General medicine

□ ④ Family medicine

□ ⑤ Other ( )

**II. Use of the emergency room (Department of Emergency Medicine)**

Please respond based on your own clinical experience with patients receiving emergency treatment for hypoglycemia.

1. How many patients with type 2 diabetes were admitted to the emergency room for hypoglycemia in the last 3 months?

( persons)

1. Please provide the percentage of patients with type 2 diabetes who were admitted to the emergency room for hypoglycemia in the last 3 months who were conscious, unconscious but breathing, or unconscious and not breathing, such that the total percentage equals 100%.

| Patients Admitted to the Emergency Room for Hypoglycemia | Percentage |
| --- | --- |
| Conscious | ( %) |
| Unconscious but breathing | ( %) |
| Unconscious and not breathing | ( %) |
| Total | 100% |

1. Please provide responses in the table below, based on ER visits (within 24 hours). For items not included in the table, please use the ‘Other’ entries. On average, what percentage (%) of the patients that you treat used the following medical resources? If the resource was used, please provide the average number of times that it was used per patient.

| **Medical Resource** | **Conscious** | **Unconscious** | |
| --- | --- | --- | --- |
|  |  | **Breathing** | **Not Breathing** |
| DW 50% ( ml) | ( %, times) | ( %, times) | ( %, times) |
| DW 10% ( ml) | ( %, times) | ( %, times) | ( %, times) |
| DW 5% ( ml) | ( %, times) | ( %, times) | ( %, times) |
| *ER tests | ( %, times) | ( %, times) | ( %, times) |
| Blood sugar test | ( %, times) | ( %, times) | ( %, times) |
| Tracheal intubation | - | ( %, times) | ( %, times) |
| Oxygen mask | - | ( %, times) | ( %, times) |
| CPR | - | ( %, times) | ( %, times) |
| Monitoring | - | ( %, times) | ( %, times) |
| Foley catheter | - | ( %, times) | ( %, times) |
| Other 1 ( ) | ( %, times) | ( %, times) | ( %, times) |
| Other 2 ( ) | ( %, times) | ( %, times) | ( %, times) |

e.g., If DW 50% was administered once for every patient, write (100%, 1 time) for DW 50%. If DW 5% was administered once each to 50% of patients with hypoglycemia, write (50%, 1 time) for DW 5%. If a resource was used irrespective of consciousness, please fill in all relevant boxes.

*ER tests:

|  | Item Measured |  | Item Measured |
| --- | --- | --- | --- |
| General chemistry tests | Sodium (Na) | Plasma protein tests | Total protein |
|  | Potassium (K) |  | Albumin |
|  | Chloride (Cl) | Urine tests | Routine Urinalysis (10) |
|  | ABGA |  | Urine sediment microscopy |
|  | Calcium |  | Uric acid |
|  | Phosphate | Lipid tests | Cholesterol |
|  | Blood urea nitrogen | Enzyme tests | AST |
|  | Creatinine |  | ALT |
|  | Total bilirubin |  | Alkaline phosphatase |
|  | Glucose |  | Amylase |
| Blood tests | Hemoglobin | Functional tests | EKG |
|  | Hematocrit | Imaging tests | Chest X-ray |
|  | White blood cell differential count | Hemorrhage/ thrombosis tests | Prothrombin Time |
|  | Red blood cell count |  | APTT |
|  | White blood cell count |  |  |
|  | Platelet Count |  |  |

ABGA, Arterial blood gas analysis; ALT, Alanine Aminotransferase; APTT, Activated Partial Thromboplastin Time; AST, Aspartate Aminotransferase; CPR, Cardiopulmonary resuscitation; DW, Dextrose in water ICU; EKG, Electrocardiography; ER, Emergency room

7-1. As with the previous question, please provide the percentages and frequencies for the following imaging tests.

| **Test** | **Conscious** | **Unconscious** | |
| --- | --- | --- | --- |
|  |  | **Breathing** | **Not Breathing** |
| Brain CT | - | ( %, times) | ( %, times) |
| MRI | - | ( %, times) | ( %, times) |
| CT + MRI | - | ( %, times) | ( %, times) |
| Total |  | 100% | 100% |

1. If any of the below tests are performed for patients with diabetes in the ER, what percentage of patients used these medical resources on average? If a resource was used, please provide the average number of times it is used per patient. Please provide the names and frequencies of additional tests or treatments in the ‘Other’ categories. Tests should be performed during the ER visit (within 24 hours).

| **Category** | **Item Measured** | **Conscious** | **Unconscious** | |
| --- | --- | --- | --- | --- |
|  |  |  | **Breathing** | **Not Breathing** |
| General chemistry tests | HbA1c  Troponin-T | ( %, times)  ( %, times) | ( %, times)  ( %, times) | ( %, times)  ( %, times) |
| Endocrinology tests | Cortisol  FT4  TSH | ( %, times)  ( %, times)  ( %, times) | ( %, times)  ( %, times)  ( %, times) | ( %, times)  ( %, times)  ( %, times) |
| Enzyme tests | CK  CK-MB | ( %, times)  ( %, times) | ( %, times)  ( %, times) | ( %, times)  ( %, times) |
| Other 1  Other 2 | ( )  ( ) | ( %, times)  ( %, times) | ( %, times)  ( %, times) | ( %, times)  ( %, times) |

e.g., If HbA1c testing was performed once for all patients, write (100%, 1 times) for HbA1c. If cortisol testing was performed once each for 50% of patients with hypoglycemia, write (50%, 1 times) for Cortisol. If a test is performed irrespective of consciousness, please fill in all relevant boxes.

Please provide the percentages below, based on your own clinical experience with patients receiving emergency treatment for hypoglycemia.

1. Among patients admitted to the emergency room for hypoglycemia, please provide the percentage of conscious patients who were discharged or hospitalized following emergency treatment.

| **Conscious Patients** | **Percentage** |
| --- | --- |
| Discharged after emergency treatment | ( %) |
| Hospitalized after emergency treatment | ( %) |
| Total | 100% |

1. Among patients admitted to the emergency room for hypoglycemia, please provide the percentage of unconscious but breathing patients who were admitted to a general ward or the ICU following emergency treatment.

| **Unconscious but Breathing Patients** | **Percentage** |
| --- | --- |
| Admitted to a general ward after emergency treatment | ( %) |
| Admitted to the ICU after emergency treatment | ( %) |
| Total | 100% |

1. Among patients admitted to the emergency room for hypoglycemia, please provide the percentage of unconscious and not breathing patients who were admitted to the ICU or following emergency treatment and the percentage who died during emergency treatment.

| **Unconscious and not Breathing Patients** | **Percentage** |
| --- | --- |
| Admitted to the ICU after emergency treatment | ( %) |
| Died during emergency treatment | ( %) |
| Total | 100% |

**(C) Primary Care Clinic Use.**

**Study of the cost of care for hypoglycemia in patients with type 2 diabetes in South Korea**

| This study aims to investigate hypoglycemia in patients with type 2 diabetes in South Korea.  In addition to verifying the incidence of hypoglycemia in patients with type 2 diabetes, the extra cost for patients with hypoglycemia will also be calculated.  The incidence of hypoglycemia by treatment type and the use of medical resources for actual treatment will be investigated.  This investigation will not be used in any form other than for research purposes, and the privacy of the information will be maintained. |
| --- |

1. **General characteristics**
2. In which region is your current place of work located?

| Metropolitan Area/Province | City/County/District |
| --- | --- |
| □ ① Seoul | □ ① ( ) City |
| □ ② ( ) Metropolitan Area | □ ② ( ) County |
| □ ③ ( ) Province | □ ③ ( ) District |

1. At which type of institution do you currently work?

□① Tertiary hospital hospital

□② Secondary hospital

□③ Primary care clinic

□④ Other ( )

1. How many years of clinical experience do you have? ( years)
2. What is your specialty?

□ ① Endocrinology

□ ② Emergency medicine

□ ③ General medicine

□ ④ Family medicine

□ ⑤ Other ( )

1. What is the average number of patients with type 2 diabetes that you treat per month?

( persons)

1. What is the sex ratio of the patients with type 2 diabetes that you treat?

| Men | Women |
| --- | --- |
| ( %) | ( %) |

1. What is the average age of the patients with type 2 diabetes that you treat?

□ ① <30 years □ ② 30–39 years

□ ③ 40–49 years □ ④ 50–59 years

□ ⑤ 60–69 years □ ⑥ 70–79 years □ ⑦ ≥80 years

1. What is the average duration of type 2 diabetes for the patients that you treat?

( years)

1. **Use of Primary care clinics**

Please respond based on your own clinical experience with patients receiving emergency treatment for hypoglycemia.

1. How many patients with type 2 diabetes visited regularly as outpatients in the last month?

( persons)

1. Of the patients with type 2 diabetes who visited regularly as outpatients in the last month, how many patients had symptoms of hypoglycemia or blood glucose levels <70mg/dL at the time of the visit?

( persons)

1. How many outpatients visited in the last month because of hypoglycemia?

( persons)

1. How many patients with hypoglycemia were transferred in the last month to the emergency room of a medical institution categorized as at least a hospital?

| Disease Course | Number of Patients Transferred to an emergency room |
| --- | --- |
| Regular patients who developed hypoglycemia | ( persons) |
| Outpatients who visited because of hypoglycemia | ( persons) |

1. Which of the following medical resources were prescribed for each outpatient, and how often was each resource prescribed, on average?

Please record the names and frequencies of additional tests or treatments in the ‘Other’ categories. Please provide detailed responses, e.g., liver function tests (AST, ALT, ALP, GGT, bilirubin), kidney function tests (Blood urea nitrogen, Serum creatinine, GFR, creatinine clearance).

|  | Medical Resource | Frequency |
| --- | --- | --- |
| Test | Blood sugar test | ( %, times)/visit |
|  | HbA1c | ( %, times)/visit |
|  | Glucose | ( %, times)/visit |
|  | Other 1 ( ) | ( %, times)/visit |
|  | Other 2 ( ) | ( %, times)/visit |
|  | Other 3 ( ) | ( %, times)/visit |
| Treatment | Dextrose in water 50% ( ml) | ( %, times)/visit |
|  | Dextrose in water 10% ( ml) | ( %, times)/visit |
|  | Other 1 ( ) | ( %, times)/visit |
|  | Other 2 ( ) | ( %, times)/visit |
|  | Other 3 ( ) | ( %, times)/visit |

ALT, Alanine Aminotransferase; ALP, Alkaline phosphatase; AST, Aspartate Aminotransferase; GFR, Glomerular filtration rate; GGT, gamma glutamyl transferase
