# Supplementary material for: Economic Burden of Hypoglycemia in Patients with Type 2 Diabetes Mellitus from Korea
Source: PLoS One. 2016 Mar 14;11(3):e0151282. doi: 10.1371/journal.pone.0151282 (PMC4790854; doi:10.1371/journal.pone.0151282)
Supplement: S1 Table — (DOCX) [file pone.0151282.s003.docx]

**S1 Table. Cost items specified in the 2014 health insurance medical care expenses.**

| **Classification** | **Division** | **Subdivision** | **Cost items** | | **Cost ($)** |
| --- | --- | --- | --- | --- | --- |
| Direct medical cost | Basic medical exam cost |  | Emergency care expense | | 41.45 |
|  |  |  | Hospitalization fee (Secondary hospitals) | | 27.48 |
|  |  |  | Hospitalization fee (Tertiary hospitals) | | 29.86 |
|  |  |  | ICU hospitalization fee (Secondary hospitals) | | 82.74 |
|  |  |  | ICU hospitalization fee (Tertiary hospitals) | | 90.39 |
|  |  |  | Second visit consultation fee (Primary care clinics) | | 8.23 |
|  |  |  | Second visit consultation fee (Secondary hospitals) | | 10.29 |
|  |  |  | Second visit consultation fee (Tertiary hospitals) | | 11.68 |
|  | Imaging diagnosis and radiation therapy expenses | Special radiographic diagnosis expense | CT (head) | | 53.98 |
|  |  |  | MRI (brain) | Secondary hospitals | 435.76 |
|  |  |  |  | Tertiary hospitals | 552.81 |
|  | Test expense | Chemical test expense | HbA1c | | 6.10 |
|  |  |  | Blood sugar test | | 0.77 |
|  |  |  | Serum glucose | | 1.02 |
|  |  |  | Serum sodium | | 0.92 |
|  |  |  | Serum potassium | | 0.99 |
|  |  |  | Serum chloride | | 0.86 |
|  |  |  | Serum calcium | | 0.94 |
|  |  |  | Serum phosphate | | 0.89 |
|  |  |  | Blood urea nitrogen | | 1.26 |
|  |  |  | Serum creatinine | | 0.99 |
|  |  |  | Total bilirubin | | 1.02 |
|  |  |  | Uric acid | | 1.06 |
|  |  |  | Troponin-T | | 9.59 |
|  |  |  | Arterial blood gas analysis (ABGA) | | 1.06 |
|  |  | Endocrine test | C-peptide | | 11.93 |
|  |  |  | Free T4 | | 10.21 |
|  |  |  | Thyroid stimulating hormone | | 13.25 |
|  |  |  | Cortisol | | 7.03 |
|  |  | Enzyme assay | Serum AST | | 1.39 |
|  |  |  | Serum ALT | | 1.35 |
|  |  |  | Alkaline phosphatase | | 1.19 |
|  |  |  | Serum amylase | | 2.41 |
|  |  |  | Serum CK | | 3.30 |
|  |  |  | Serum CK-MB | | 9.02 |
|  |  | Function test | Electrocardiography | | 4.06 |
|  |  | Hematological test | Hematocrit | | 0.65 |
|  |  |  | Hemoglobin | | 0.99 |
|  |  |  | Red blood cell count | | 0.65 |
|  |  |  | White blood cell differential count | | 1.49 |
|  |  |  | Platelet count | | 0.77 |
|  |  |  | White blood cell count | | 0.65 |
|  |  | Hemorrhage and thrombus test | APTT | | 2.63 |
|  |  |  | Prothrombin time | | 1.63 |
|  |  | Imaging test | Chest X-ray | | 4.69 |
|  |  | Lipid test | Determination of cholesterol | | 1.30 |
|  |  | Plasma protein test | Albumin | | 1.33 |
|  |  |  | Total Protein | | 1.05 |
|  |  | Urine test | Routine urinalysis | | 1.67 |
|  |  |  | Urine sediment microscopy | | 0.66 |
|  | Treatment |  | Cardiopulmonary resuscitation | | 42.29 |
|  |  |  | Dextrose in water 5% 500 mL | | 1.05 |
|  |  |  | Dextrose in water 5% 1000 mL | | 1.38 |
|  |  |  | Dextrose in water 10% 500 mL | | 1.14 |
|  |  |  | Dextrose in water 10% 1000 mL | | 1.43 |
|  |  |  | Dextrose in water 50% 50 mL | | 1.73 |
|  |  |  | Dextrose in water 50% 100 mL | | 1.12 |
|  |  |  | Foley catheter | | 7.67 |
|  |  |  | Intubation | | 16.50 |
|  |  |  | Monitoring | | 14.03 |
|  |  |  | Normal saline 1000 mL | | 1.08 |
|  |  |  | Oxygen mask | | 4.96 |

ALT, Alanine Aminotransferase; APTT, Activated Partial Thromboplastin Time; AST, Aspartate Aminotransferase; CK, Creatine Kinase; CK-MB, Creatine Kinase-Myocardial Band; CT, Computerized Tomography; ICU, Intensive Care Unit; MRI, Magnetic Resonance Imaging
