# Supplementary material for: Economic Burden of Hypoglycemia in Patients with Type 2 Diabetes Mellitus from Korea
Source: PLoS One. 2016 Mar 14;11(3):e0151282. doi: 10.1371/journal.pone.0151282 (PMC4790854; doi:10.1371/journal.pone.0151282)
Supplement: S2 Table — (DOCX) [file pone.0151282.s004.docx]

**S2 Table.** **Characteristics of physicians and patients with type 2 diabetes mellitus.**

| Variable | | | Secondary and tertiary hospitals | | | | |  | Primary care clinics | |
| --- | --- | --- | --- | --- | --- | --- | --- | --- | --- | --- |
|  |  |  | **Endocrinologists** |  | | **Emergency**  **physicians** | |  | **Primary care physicians** | |
| Physician’s number | | | 40 |  | | 20 | |  | 30 | |
| Region, n (%) | | |  |  | |  | |  |  | |
|  | Seoul | | 13 (32.5) |  | | 8 (40) | |  | 13 (43.3) | |
|  | Gyeonggi | | 13 (32.5) |  | | 5 (25) | |  | 3 (10) | |
|  | Daegu | | 3 (7.5) |  | | 1 (5) | |  | 3 (10) | |
|  | Busan | | 3 (7.5) |  | | 2 (10) | |  | 3 (10) | |
|  | Incheon | | 2 (5) |  | | 1 (5) | |  | 2 (6.7) | |
|  | Daejeon | | 2 (5) |  | | 1 (5) | |  | 3 (10) | |
|  | Gwangju | | 2 (5) |  | | 2 (10) | |  | 3 (10) | |
|  | Jeonbuk | | 1 (2.5) |  | | - | |  | - | |
|  | Chungbuk | | 1 (2.5) |  | | - | |  | - | |
| Physicians’ clinical experience (years), mean (SD) | | | | | | | | | | |
|  |  | | 13.9 (7.13) |  | 12.7 (6.1) | | |  | 20.0 (4.6) | |
| Monthly number of treated T2DM patients per physician, mean (SD) | | | | | | | | | | |
|  |  | | 650.0 (227.9) |  |  | |  |  | 433.7 (209.0) | |
|  |  | |  |  | 82.0 (68.1) | | | |  | |
| Sex ratio of T2DM patients, % | | | | |  | |  |  |  |  |
|  | Male | | 45.0 |  |  | |  |  | 43.5 | |
|  | Female | | 55.0 |  |  | |  |  | 56.5 | |
|  | |  | 57.2 (5.1) |  |  | |  |  | 56.3 (4.0) | |
|  | |  | 7.7 (2.2) |  |  | |  |  | 6.9 (3.0) | |

SD; Standard Deviation; T2DM, Type 2 Diabetes Mellitus
