# Supplementary material for: Economic Burden of Hypoglycemia in Patients with Type 2 Diabetes Mellitus from Korea
Source: PLoS One. 2016 Mar 14;11(3):e0151282. doi: 10.1371/journal.pone.0151282 (PMC4790854; doi:10.1371/journal.pone.0151282)
Supplement: S3 Table — (A) Department of Endocrinology in secondary and tertiary hospitals. (B) Department of Emergency Medicine in secondary and tertiary hospitals. (C) Primary care clinics. (DOCX) [file pone.0151282.s005.docx]

**S3 Table. Results of the study of medical resources usage.**

**(A) Department of Endocrinology in secondary and tertiary hospitals.**

| Classification | Medical resources | Prescription rate | Prescription frequency (Times) | Mean (SD) |
| --- | --- | --- | --- | --- |
| Use of outpatient care | | | | |
| Test | Blood sugar test | 90 % | 1.1 |  |
|  | Glucose | 60 % | 0.9 |  |
|  | HbA1c | 60 % | 1 |  |
| Treatment | Dextrose in water 50% 50mL | 4 % | 1 |  |
|  | Dextrose in water 50% 100mL | 5 % | 1.3 |  |
|  | Dextrose in water 10% 500mL | 7 % | 1 |  |
|  | Dextrose in water 10% 1000mL | 1 % | 1 |  |
| Hospitalization in general ward | | | | |
| Others | Hospitalization period |  |  | 5.0 (1.9) |
| Test | Blood sugar test prescription frequency (1 day) |  |  | 5.1 (1.3) |
|  | C-peptide (per hospitalization) |  |  | 1.1 (0.5) |
|  | Lab test (per hospitalization) |  |  | 1.9 (1.1) |
|  | HbA1c | 93 % | 1 |  |
|  | CK | 38 % | 1 |  |
|  | CK-MB | 32 % | 1 |  |
|  | Troponin-T | 31 % | 1 |  |
|  | Free T4 | 58 % | 1 |  |
|  | Thyroid stimulating hormone | 60 % | 1 |  |
|  | Cortisol | 38 % | 1 |  |
| Treatment | Education on diabetes (per hospitalization) |  |  | 0.9 (0.4) |
|  | Normal saline 1000mL (1 day) |  |  | 0.7 (0.7) |
| Hospitalization in ICU | | | | |
| Others | ICU hospitalization period |  |  | 5.3 (4.2) |
| Test | Lab test (per ICU hospitalization) |  |  | 5.2 (3.7) |
| Treatment | Foley catheter | 59 % | 1.8 |  |
|  | Cardiopulmonary resuscitation | 4 % | 1 |  |
|  | Dextrose in water 50% 50mL | 28 % | 1.3 |  |
|  | Dextrose in water 50% 100mL | 28 % | 1.9 |  |
|  | Dextrose in water 10% 500mL | 19 % | 1.4 |  |
|  | Dextrose in water 10% 1000mL | 44 % | 2.9 |  |
|  | Dextrose in water 5% 500mL | 3 % | 2.3 |  |
|  | Dextrose in water 5% 1000mL | 49 % | 1 |  |
|  | Intubation | 11 % | 1 |  |
|  | Monitoring | 90 % | 1.3 |  |
|  | Oxygen mask | 36 % | 1.1 |  |

**(B) Department of Emergency Medicine in secondary and tertiary hospitals.**

| Classification | Medical resources | | Prescription rate | Prescription frequency |
| --- | --- | --- | --- | --- |
| Conscious patient | | | | |
| Test | | Blood sugar test | 95 % | 2.2 |
|  |  | CK | 53 % | 1 |
|  |  | CK-MB | 54 % | 1 |
|  |  | Troponin-T | 36 % | 1 |
|  |  | Cortisol | 5 % | 1 |
|  |  | ER Lab test | 100 % | 1 |
|  |  | Free T4 | 6 % | 1 |
|  |  | Thyroid stimulating hormone | 6 % | 1 |
|  |  | HbA1c | 75 % | 1 |
| Treatment | | Dextrose in water 50% 50ml | 36 % | 1 |
|  |  | Dextrose in water 50% 100ml | 27 % | 1 |
|  |  | Dextrose in water 10% 500ml | 51 % | 1 |
|  |  | Dextrose in water 10% 1000ml | 22 % | 1 |
|  |  | Dextrose in water 5% 500ml | 3 % | 1 |
|  |  | Dextrose in water 5% 1000ml | 10 % | 1 |
| Unconscious but self-breathing patient | | | | |
| Imaging diagnosis and radiation therapy | | CT | 65 % |  |
|  |  | CT & MRI | 26 % |  |
|  |  | MRI | 9 % |  |
| Test | | Blood sugar test | 100 % | 2.2 |
|  |  | CK | 75 % | 1.1 |
|  |  | CK-MB | 71 % | 1.1 |
|  |  | Troponin-T | 53 % | 1 |
|  |  | Cortisol | 9 % | 1 |
|  |  | ER Lab test | 100 % | 1.1 |
|  |  | Free T4 | 10 % | 1 |
|  |  | Thyroid stimulating hormone | 10 % | 1 |
|  |  | HbA1c | 70 % | 1 |
| Treatment | | Foley catheter | 19 % | 1 |
|  |  | Cardiopulmonary resuscitation | - | - |
|  |  | Dextrose in water 50% 50ml | 64 % | 1.2 |
|  |  | Dextrose in water 50% 100ml | 31 % | 1.7 |
|  |  | Dextrose in water 10% 500ml | 43 % | 1 |
|  |  | Dextrose in water 10% 1000ml | 23 % | 1 |
|  |  | Dextrose in water 5% 500ml | 3 % | 1 |
|  |  | Dextrose in water 5% 1000ml | 4 % | 1 |
|  |  | Intubation | 7 % | 1 |
|  |  | Monitoring | 86 % | 1 |
|  |  | Oxygen mask | 37 % | 1.1 |
| Unconscious and non self-breathing patient | | | | |
| Imaging diagnosis and radiation therapy | | CT | 69 % |  |
|  |  | CT & MRI | 18 % |  |
|  |  | MRI | 13 % |  |
| Test | | Blood sugar test | 100 % | 2.7 |
|  |  | CK | 93 % | 1 |
|  |  | CK-MB | 93 % | 1 |
|  |  | Troponin-T | 80 % | 1 |
|  |  | Cortisol | 24 % | 1 |
|  |  | ER Lab test | 100 % | 1.2 |
|  |  | Free T4 | 26 % | 1 |
|  |  | Thyroid stimulating hormone | 26 % | 1 |
|  |  | HbA1c | 77 % | 1 |
| Treatment | | Foley catheter | 84 % | 1 |
|  |  | Cardiopulmonary resuscitation | 18 % | 1 |
|  |  | Dextrose in water 50% 50ml | 67 % | 1.3 |
|  |  | Dextrose in water 50% 100ml | 30 % | 1 |
|  |  | Dextrose in water 10% 500ml | 33 % | 1 |
|  |  | Dextrose in water 10% 1000ml | 3 % | 1 |
|  |  | Dextrose in water 5% 500ml | - | - |
|  |  | Dextrose in water 5% 1000ml | 3 % | 1 |
|  |  | Intubation | 100 % | 1 |
|  |  | Monitoring | 100 % | 1 |
|  |  | Oxygen mask | 33 % | 1 |

**(C) Primary care clinics.**

| Classification | Medical resources | Prescription rate | Prescription frequency |
| --- | --- | --- | --- |
| Prescription | Dextrose in water 50% 50mL | <0.1 % | 1.0 |
|  | Dextrose in water 50% 100mL | 4 % | 1.0 |
|  | Dextrose in water 10% 500mL | 10 % | 1.0 |
|  | Dextrose in water 10% 1000mL | 0 % | - |
| Test | Blood sugar test | 77 % | 1.0 |
|  | Serum glucose | 56 % | 0.8 |
|  | HbA1c | 43 % | 0.8 |

CK, Creatine Kinase; CK-MB, Creatine Kinase-Myocardial Band; CT, Computerized Tomography; ER, Emergency room; ICU, Intensive Care Unit; MRI, Magnetic Resonance Imaging
