# Supplementary material for: Economic Burden of Hypoglycemia in Patients with Type 2 Diabetes Mellitus from Korea
Source: PLoS One. 2016 Mar 14;11(3):e0151282. doi: 10.1371/journal.pone.0151282 (PMC4790854; doi:10.1371/journal.pone.0151282)
Supplement: S4 Table — (DOCX) [file pone.0151282.s006.docx]

**S4 Table. Hypoglycemia treatment costs in type 2 diabetes mellitus patients who visited secondary and tertiary hospitals**

| Type | Cost ($)ª | | | | |
| --- | --- | --- | --- | --- | --- |
|  | **Total** | **Outpatient** | **ER** | **Hospitalization** | **ICU** |
| A | 17.28 | 17.28  (4.40) |  |  |  |
| B | 368.96 | 17.28  (4.40) |  | 351.69  (117.31) |  |
| C | 17.28 | 17.28  (4.40) |  |  |  |
| D | 368.96 | 17.28  (4.40) |  | 351.69  (117.31) |  |
| E | 123.80 |  | 123.80  (10.29) |  |  |
| F | 475.48 |  | 123.80  (10.29) | 351.69  (117.31) |  |
| G | 690.61 |  | 338.92  (65.98) | 351.69  (117.31) |  |
| H | 1,577.75 |  | 338.92  (65.98) | 351.69  (117.31) | 887.14  (492.89) |
| I | 1,857.09 |  | 618.26  (100.38) | 351.69  (117.31) | 887.14  (492.89) |
| J | 1,226.07 |  | 338.92  (65.98) |  | 887.14  (492.89) |
| K | 1,505.41 |  | 618.26  (100.38) |  | 887.14  (492.89) |
| L | 618.26 |  | 618.26  (100.38) |  |  |

**ª**Costs are expressed as mean (standard deviation)

ER, Emergency Room; ICU, Intensive Care Unit
